# Reconstruction of an orbital fracture in a mare using a 3D‐printed patient‐specific implant

**DOI:** 10.1111/vsu.70050

**Published:** 2025-10-30

**Authors:** Jennifer Gernhardt, Peter Böttcher, J. Corinna Eule, Kathrin Mählmann, Eva Müller, Christoph J. Lischer

**Affiliations:** ^1^ Equine Clinic, Freie Universität Berlin Berlin Germany; ^2^ Small Animal Clinic, Freie Universität Berlin Berlin Germany; ^3^ Unit for Ophthalmology, Veterinary Hospital, Freie Universität Berlin Berlin Germany

## Abstract

**Objective:**

To describe surgical repair of an orbital wall fracture communicating with the caudal maxillary sinus using a three dimensional (3D)‐printed, patient‐specific implant (PSI).

**Study design:**

Case report.

**Animal:**

A 25‐year‐old Haflinger mare (370 kg).

**Methods:**

The mare presented with severe chemosis and emphysema of the left upper and lower eyelid of 2‐weeks duration due to a fracture of the ventral floor and inner wall of the left orbit that communicated with the caudal maxillary sinus. Computed tomographic (CT) data were used to design a PSI replicating the inner surface of the unfractured orbit. The data were converted to an STL file and 3D‐printed using polylactic acid (PLA) filament. Surgical access was obtained via a concho‐frontal sinus flap combined with sinoscopy. To reconstruct and seal the orbit, the PSI was lined with porcine small intestinal submucosa and secured to the inner orbital wall using two 3.0 mm titanium locking screws and synthetic, non‐absorbable transosseous fiber loops.

**Results:**

The emphysema resolved postoperatively, and ocular function was unrestricted by the implant. Infection developed subcutaneously adjacent to the transosseous suture fixation at the maxillary bone but resolved with conservative management. Follow‐up CT at 11 weeks and owner reports after 18 months confirmed a favorable cosmetic and functional outcome, with only minimal residual ptosis of the left upper eyelid.

**Conclusion:**

Application of a PSI axially to the left orbit successfully reconstructed the orbital wall and resolved the communication with the caudal maxillary sinus.

**Clinical significance:**

Patient‐specific implants represent a novel surgical option for the repair of complex orbital wall fractures in horses.

## 1 INTRODUCTION

The bony orbit of horses consists of the frontal, lacrimal, temporal, and zygomatic bones.^1^ The outer portions of the orbit form the orbital rim, which is composed of the frontal process of the zygomatic bone as well as the zygomatic processes of the frontal and temporal bones.^2^ Caudal to the orbital rim lies the inner orbital wall, which consists of the lacrimal, sphenoid, and palatine bones. The rostral aspect of the orbital floor is formed by the temporal process of the zygomatic bone. In contrast to the solid orbital rim, the bones of the orbital floor and wall are relatively thin. The remaining ventral orbit is shaped by the periorbital fascia, orbital fat, and the pterygoid muscle.^3^

Horses have been reported to sustain orbital fractures as a result of direct trauma, most commonly associated with rearing in confined spaces, falling, or colliding with stationary objects.^2^, ^4^, ^5^, ^6^ Kicks from other horses are also a common cause, especially during turnout on pasture.^5^, ^6^, ^7^ The equine orbit is relatively exposed compared to other species, and the thin medial orbital wall in particular predisposes it to fracture when subjected to blunt force trauma.^1^ Depending on the direction and intensity of the force, orbital fractures may occur with adjacent craniofacial fractures.^6^

Orbital fractures can heal without surgical intervention if they are non‐displaced, stable, and closed.^6^, ^8^ Surgical debridement is recommended for open or contaminated fractures. Displaced and unstable fractures of the solid orbital rim may require reconstruction and fixation with implants.^1^ In contrast, fractures of the inner wall and ventral orbital floor require reconstruction because retrobulbar soft tissues can herniate through the defect into the adjacent sinus, resulting in globe displacement, impaired ocular motility, and persistent emphysema.^7^, ^9^, ^10^ The prognosis depends on how complex the fracture is and how much damage the eye has sustained.^6^

Previous case reports have described the fixation of fractures of the inner orbital wall by inserting tissue expander devices into the concho‐frontal sinus or by using intrasinus bolstering with Foley catheter balloons.^7^, ^9^ Due to the complex topography of the equine orbital region and the frequent occurrence of comminuted fracture patterns, three dimensional (3D)‐printed models of orbital depression fractures have been utilized to facilitate surgical planning and intraoperative orientation.^9^ Despite the availability of 3D‐renderings of the skull, 3D‐printed models were found to be helpful in determining the orientation of the fracture fragments.^9^ The advent of 3D‐printing has opened new possibilities for implant manufacturing and is increasingly being adopted in veterinary medicine.^11^, ^12^, ^13^ In human medicine, patient‐specific implants are considered the most customizable option for restoring the facial skeleton symmetrically in oculofacial surgery.^14^

The aim of the study was to report the fixation of a complex inner orbital wall fracture in an equine patient using a 3D‐printed, patient‐specific implant (PSI). To the best of the authors' knowledge, this is the first report of such a surgical repair. We hypothesized that fracture repair with a PSI represents a feasible, surgical option for orbital wall fractures with communication with the sinus system.

## 2 MATERIALS AND METHODS

### 2.1 History and clinical findings

A 25‐year‐old Haflinger mare (370 kg), maintained on pasture with other horses, was referred to the Equine Hospital of the Free University of Berlin for a computed tomography (CT) scan due to severe chemosis and emphysema of the left upper and lower eyelid. Two weeks prior to referral, the mare had sustained a suspected traumatic injury resulting in swelling of the left upper and lower eyelid and a superficial wound over the left facial crest, which had been examined and treated conservatively by the referring veterinarian. On initial examination, fluorescein staining revealed several small abrasions of the left cornea, which were treated topically with a colloidal silver solution. There was no improvement of the swelling of the left upper and lower eyelid following conservative management.

Upon admission to the clinic, the mare was bright, alert, and responsive, with all vital parameters within normal limits. Feed intake and fecal output appeared unaffected, and complete blood count, hematocrit, and total protein values were within normal reference ranges. The left eyelids exhibited marked swelling with crepitus on palpation, consistent subcutaneous emphysema. The conjunctiva, particularly that of the upper eyelid, was severely chemotic, hyperemic, and protruding. Examination of the cranial nerves revealed no abnormalities. The dazzle reflex in the left eye and the consensual pupillary light reflex in the right eye were present. A complete ophthalmologic examination of the right eye, including assessment of the pupillary light reflexes, anterior chamber, and fundus, revealed no abnormalities.

### 2.2 Diagnostic imaging

Transpalpebral ultrasonographic examination of the internal ocular structures of the left eye confirmed retro‐ and periocular emphysema. All ocular structures were within normal limits, suggesting intact visual function.

Radiographic examination of the head (latero‐lateral projection) revealed a fracture line involving the ventral orbit. However, there was no visible fluid line in the sinuses.

**FIGURE 1:**
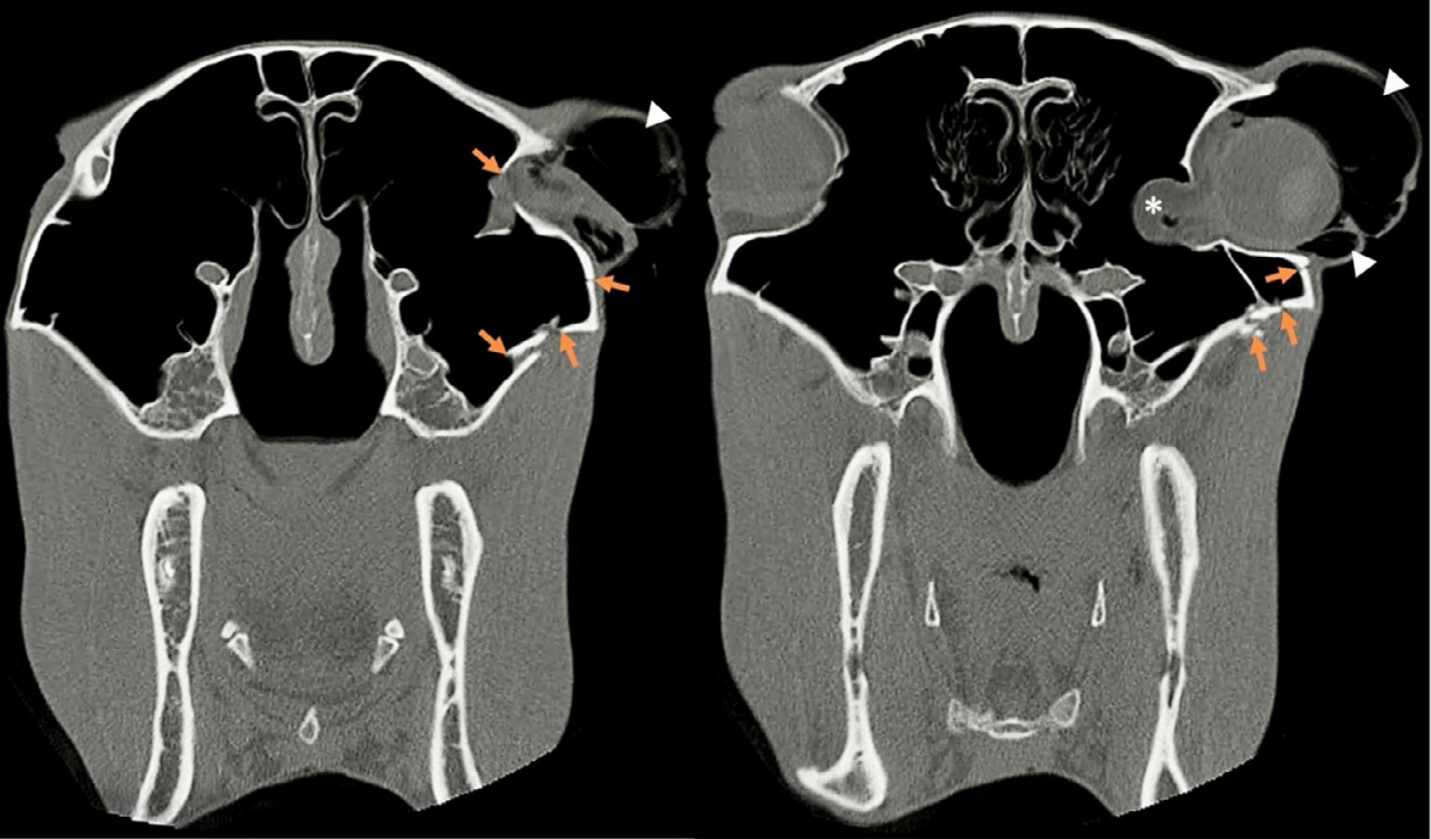
Preoperative transverse computed tomography of the skull. Note the multiple fracture lines at the inner orbital wall and around the facial crest (arrows) and the prolapse of orbital tissue into the caudal maxillary sinus secondary to a fracture of the inner orbital wall (*). A conspicuous hypoattenuating accumulation of air is present within the upper and lower eyelids (arrowheads).

Standing transverse CT imaging (Aquilion, Canon Medical Systems, Amstelveen, Netherlands), with a field‐of‐view of 512 × 512 pixels, 135 kV, 370 mA, 1 mm slice thickness and helical scanning, revealed a comminuted fracture of the left temporal process of the zygomatic bone and the left lacrimal bone, affecting the left ventral floor and inner orbital wall (Figure 1). Comminution of the inner orbital wall resulted in a defect measuring 2.1 cm in length and 3.3 cm in width, through which retrobulbar soft tissue bulged into the left caudal maxillary sinus. In addition, three small, isolated bone fragments from the inner orbital wall were displaced into the sinus. There was severe accumulation of gas in the retrobulbar area of the left eye, the upper and lower eyelid, the left pterygopalatine fossa, the left infraorbital canal at the level of the maxillary fornix, and the subcutis over the left temporalis muscle. Furthermore, multiple horizontal fracture lines were evident in the left lateral maxillary bone, both dorsal and ventral to the facial crest. These fractures separated the facial crest into rostral and caudal bone fragments. Finally, a rostral bone fragment (1.9 × 1.3 cm) and a middle fragment (5.2 × 1.2 cm) were mildly displaced into the left maxillary sinus ventral to the facial crest. There were no signs of sinusitis or associated callus formation.

### 2.3 Conservative treatment

As the fracture communicated with the caudal maxillary sinus, it was categorized as open, and infection could not be ruled out. The mare received antimicrobials (15 mg/kg BW amoxicillin IV [Belamox, bela‐pharm GmbH & Co. KG, Vechta, Germany] every 8 h); 6.6 mg/kg BW gentamicin IV (Genta, CP‐Pharma, Burgdorf, Germany) every 24 h; 15 mg/kg BW metronidazole orally (Metrotab vet., CP‐Pharma) every 8 h and analgesia (1.1 mg/kg BW flunixin‐meglumine IV [Flunidol, CP‐Pharma] every 12 h). After 8 days, antimicrobials were transitioned to oral administration (25 mg/kg BW sulfadimethoxin, 5 mg/kg BW trimethoprim orally, Sulfadimethoxin + Trimethoprim 50%, Serumwerk Bernburg AG, Bernburg, Germany) every 12 h. The protruding conjunctiva was treated locally with a nourishing ophthalmic ointment containing vitamin A and dexpanthenol (Vitamycin, CP‐Pharma) applied every 12 h. As there was no improvement after 7 days of conservative management, and the subcutaneous emphysema as well as the protruding conjunctiva persisted, a surgical plan was formulated.

### 2.4 Presurgical planning and patient‐specific implant manufacturing

A decision was made to reconstruct the orbital wall defect using an anatomically contoured shell implant, which was introduced via the sinus cavity. This approach allowed repositioning of the prolapsed orbital soft tissues while sealing the defect.

Implant design was based on transverse CT images of the skull and a virtual 3D model of the left and right orbital regions, including the soft tissues of the left orbit. These were created using an anatomical analysis and planning software (Mimics Innovation Suite, Materialize, Leuven, Belgium). The right, unaffected orbit was mirrored and matched onto the left, fractured side to serve as a template for the implant design. A surface area extending at least 1 cm beyond the fracture margins was manually delineated from the inner wall of the mirrored right orbit and augmented to a thickness of 1.5 mm to serve as the implant base.

To enhance vascular integration, multiple fenestrations, each 1 cm in diameter, were perforated into the implant. Two reinforced screw blocks were added for fixation with 3.0 mm locking screws, and three eyelets were incorporated along the ventral and rostroventral margins to anchor suture loops.

The implant was fabricated using fused deposition modeling with a Bambu Lab X1 Carbon 3D printer (Bambu Lab, Shenzhen, China), at a layer height of 0.1 mm and 100% infill. A bio‐based, high‐performance PLA (polylactic acid) filament (GreenTEC Pro, Extrudr, Gumperling, Austria; diameter 1.75 mm) was used as printing material. This material is known to be biocompatible and suitable for steam sterilization.^15^ The PSI was sterilized at 121°C at 1.1 bar for 30 min.

### 2.5 Surgery

**FIGURE 2:**
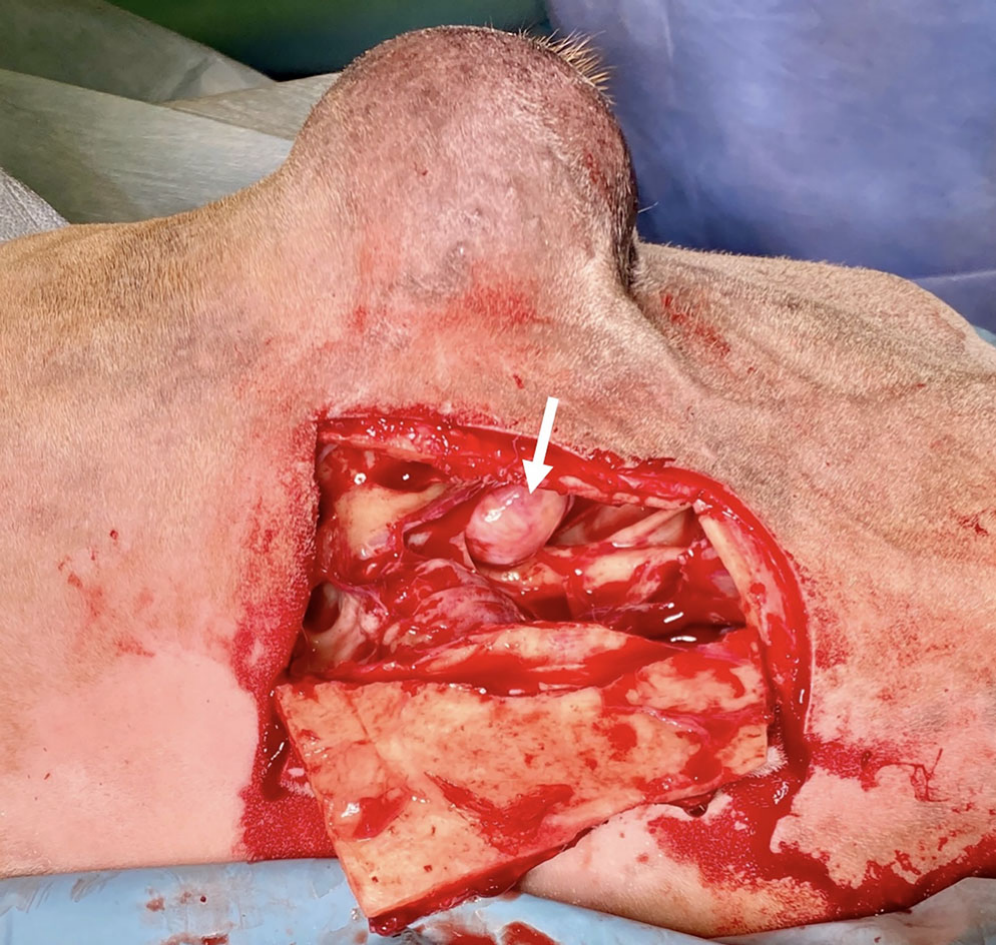
Intraoperative photograph (frontal view; left side of the horse is at the top of the image, rostral is to the right). Marked emphysema of the upper eyelid is evident. The caudal maxillary sinus was accessed via a frontomaxillary opening created by elevating a frontonasal bone flap. Orbital soft tissue is seen protruding through the fracture in the inner orbital wall from the left retrobulbar space (white arrow).

Food was withheld for 5 h prior to surgery. After appropriate sedation of the horse (0.06 mg/kg BW acepromazine IM, Tranquisol, CP‐Pharma, Burgdorf, Germany and 0.8 mg/kg BW xylazine IV, Xylavet, CP‐Pharma) and administration of analgesia (0.025 mg/kg BW butorphanol IV, Butorgesic, CP‐Pharma and 1.1 mg/kg BW flunixin‐meglumine IV, Flunidol, CP‐Pharma), general anesthesia was induced (0.05 mg/kg BW diazepam IV, Solupan, Dechra Veterinary Products GmbH, Aulendorf, Germany and 2.2 mg/kg BW ketamine IV, Ketamin, CP‐Pharma) with the horse positioned between a wall and a swing gate. A person supported the horse's head to ensure safe sternal recumbency before repositioning into lateral recumbency. The mare was subsequently intubated with an endotracheal tube and placed in right lateral recumbency. Anesthesia was maintained with isoflurane (Iso‐vet, Dechra Veterinary Products GmbH) in oxygen (minimum alveolar concentration 0.7%) and constant rate infusion (0.8 mg/kg/h xylazine IV, Xylavet, CP‐Pharma). The dorsal and the left lateral aspects of the head were aseptically prepared, and routine surgical draping was applied. After skin incision, a standard frontonasal bone flap measuring 7 cm rostrocaudally and 10 cm laterally, was made with an oscillating saw (Colibri II, DePuy Synthes, Johnson & Johnson MedTech).^16^ The frontomaxillary opening was extended to allow access to the axial aspect of the left orbit. The fracture site was clearly visible, and the prolapsed soft tissue was easily reduced (Figure 2).

To achieve an airtight seal over the orbital fracture site, the surface of the PSI that was in contact with the fractured orbital wall was covered with two layers of porcine small intestinal submucosa (SIS) (Vetrix BioSIS, Cumming, Georgia). This provided both a seal and compensation for minor discrepancies between the PSI and the reduced orbital contour. The implant was positioned axially to the orbit and fixed dorsally using two 3.0 mm titanium locking screws (KLS, KYON, Zurich, Switzerland) with a length of 12 and 14 mm. During screw tightening, the caudal screw hole in the implant fractured. Therefore, a new hole was drilled in the implant slightly rostrally to the fractured screw hole, and the screw was securely placed.

**FIGURE 3:**
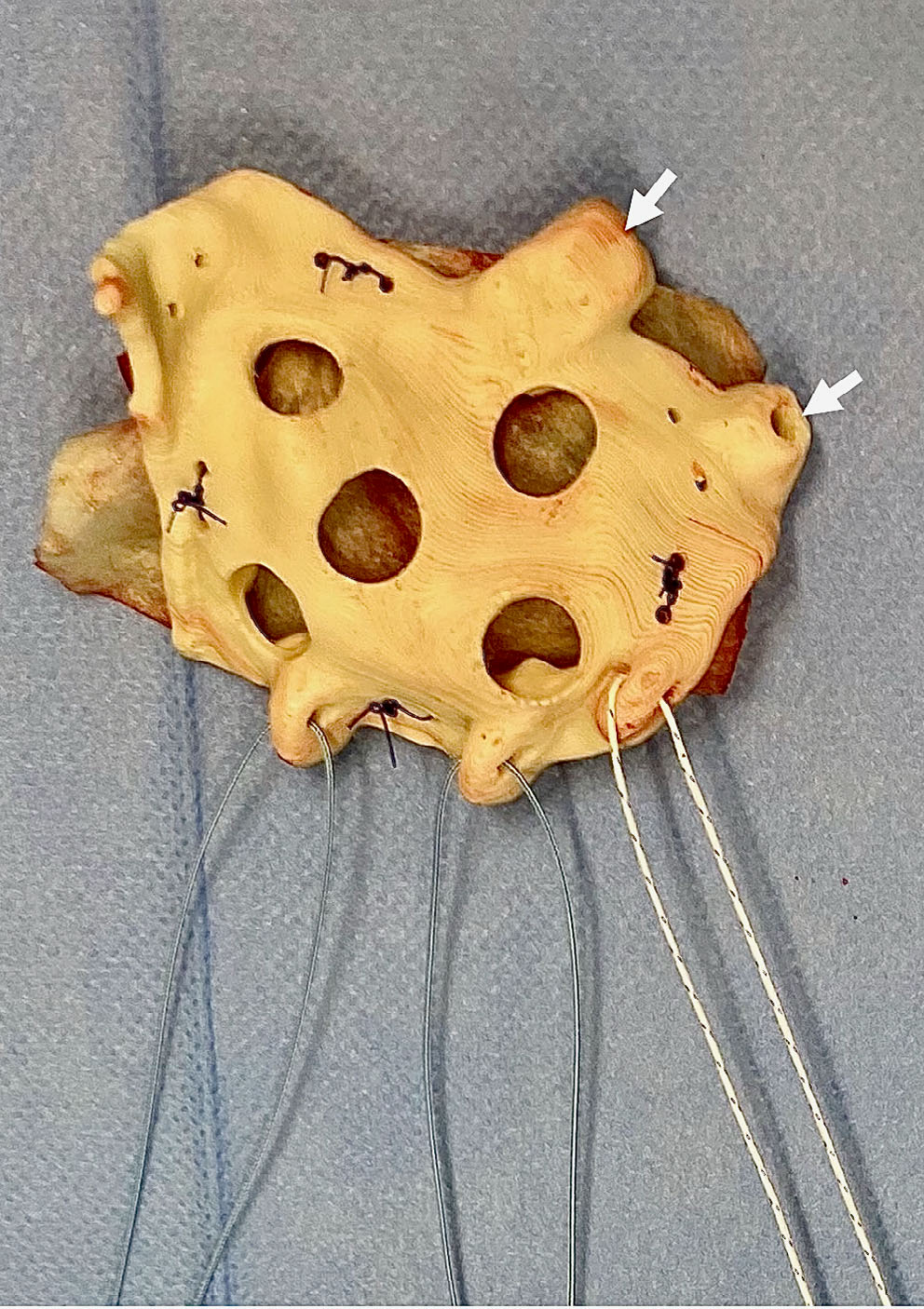
Medial view of the patient‐specific implant (PSI) with small intestinal submucosa (SIS) attached to the side facing the fracture. Arrows indicate the reinforced screw blocks designed for fixation with 3.0 mm locking screws. Fiber loops are threaded through preassembled eyelets along the ventral margin of the PSI.

**FIGURE 4:**
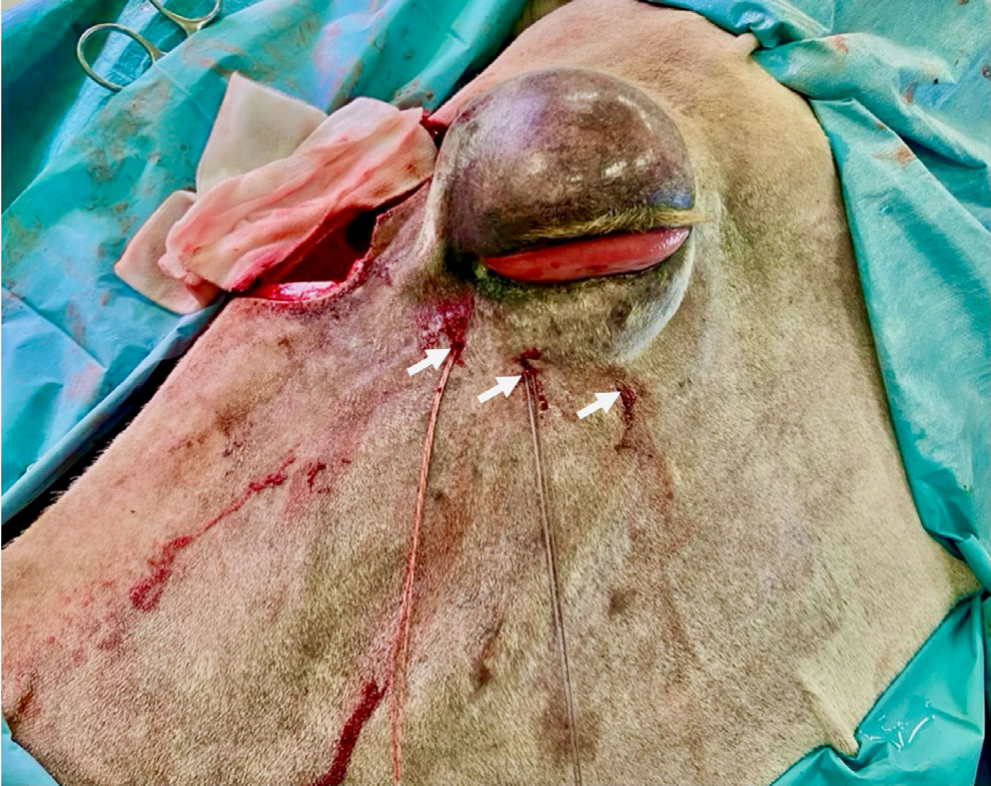
Intraoperative photograph showing severe chemosis and emphysema of the left eyelids. The arrows indicate the transosseous bone tunnels. Two of the three toggle sutures are visible ventral to the affected area.

**FIGURE 5:**
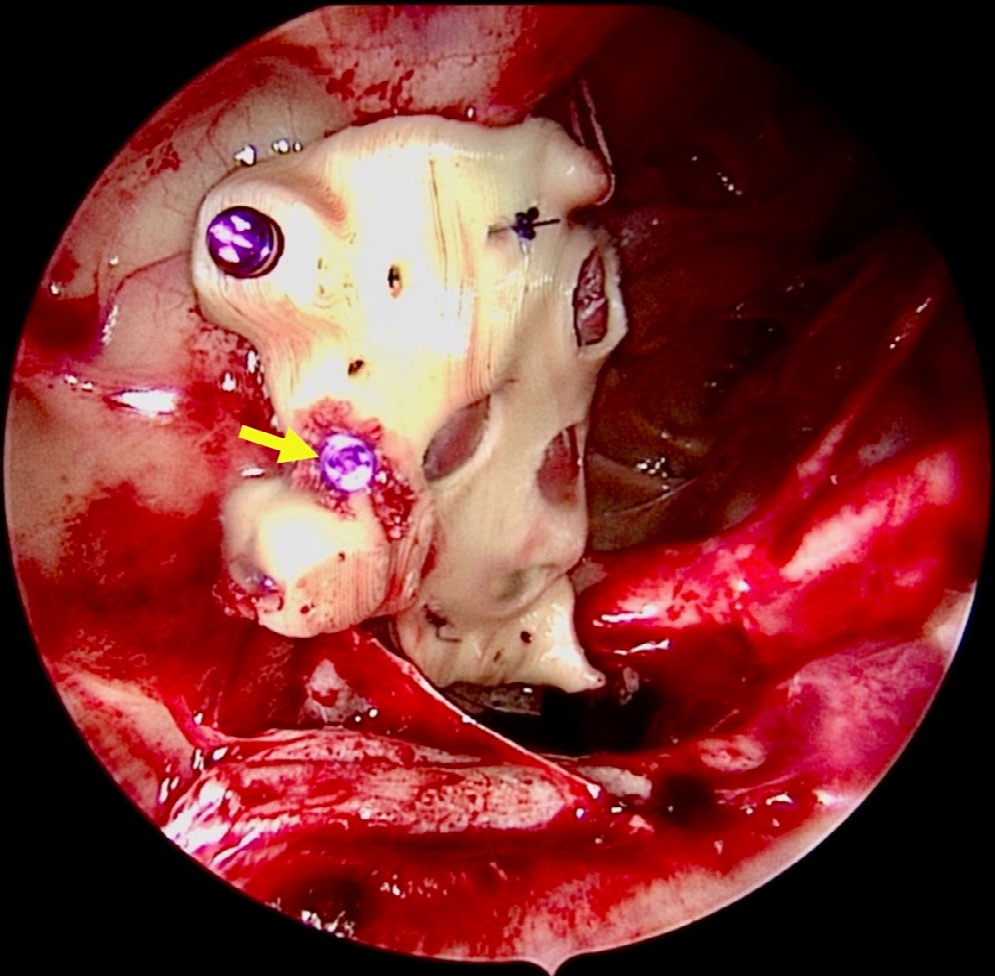
Sinuscopic view of the left caudal maxillary sinus. The patient‐specific implant (PSI) is secured with screws at its dorsal margin, axial to the left orbit. The arrow indicates an additional screw inserted following fracture of the caudal screw block.

Ventral screw fixation was not feasible due to limited access through the concho‐frontal sinus fenestration. Instead, three preattached synthetic, non‐absorbable ultrahigh molecular weight polyethylene fiber loops (FiberWire, Suture size 5, Arthrex VetSystems, Munich, Germany) were anchored to flanges located at the ventral and rostroventral margins of the PSI (Figure 3). Following dorsal fixation of the implant, three stab incisions were made approximately 3 cm ventral to the lower eyelid margin. Through these incisions, three 3.0‐mm transosseous bone tunnels were drilled from lateral to medial into the zygomatic bone (Figure 4). The fiber loops were then passed through the tunnels from within the sinus, grasped externally, and pulled to the skin surface. This ensured that the PSI was secured against the axial aspect of the fractured orbit under endoscopic guidance (Hopkins, Karl Storz, Tuttlingen, Germany: 5.5 mm, 30° optic, 4 mm in diameter, 18 cm in length) (Figure 5). The suture loops were securely anchored subcutaneously on the lateral aspect of the zygomatic bone by tying each loop over a separate suture button (Suture Button, Arthrex VetSystems). The skin over the suture buttons and the bone flap was closed in a simple interrupted suture pattern (Prolene USP 2/0, Ethicon, Johnson & Johnson MedTech, New Brunswick, New Jersey). The excessive air trapped in the upper eyelid was removed by aspiration with an injection needle (20 gauge × 1 ½″ 0.90 × 40 mm). The wounds were protected with sterile adhesive dressing (Mepilex Border Flex Oval, Mölnlycke Health Care GmbH, Dusseldorf, Germany), which was kept in place using adhesive tape (Optiplaste‐C, BSN medical GmbH, Hamburg, Germany). The total surgical time was approximately 3 h, and recovery, assisted with head and tail ropes, was uneventful.

## 3 RESULTS

**FIGURE 6:**
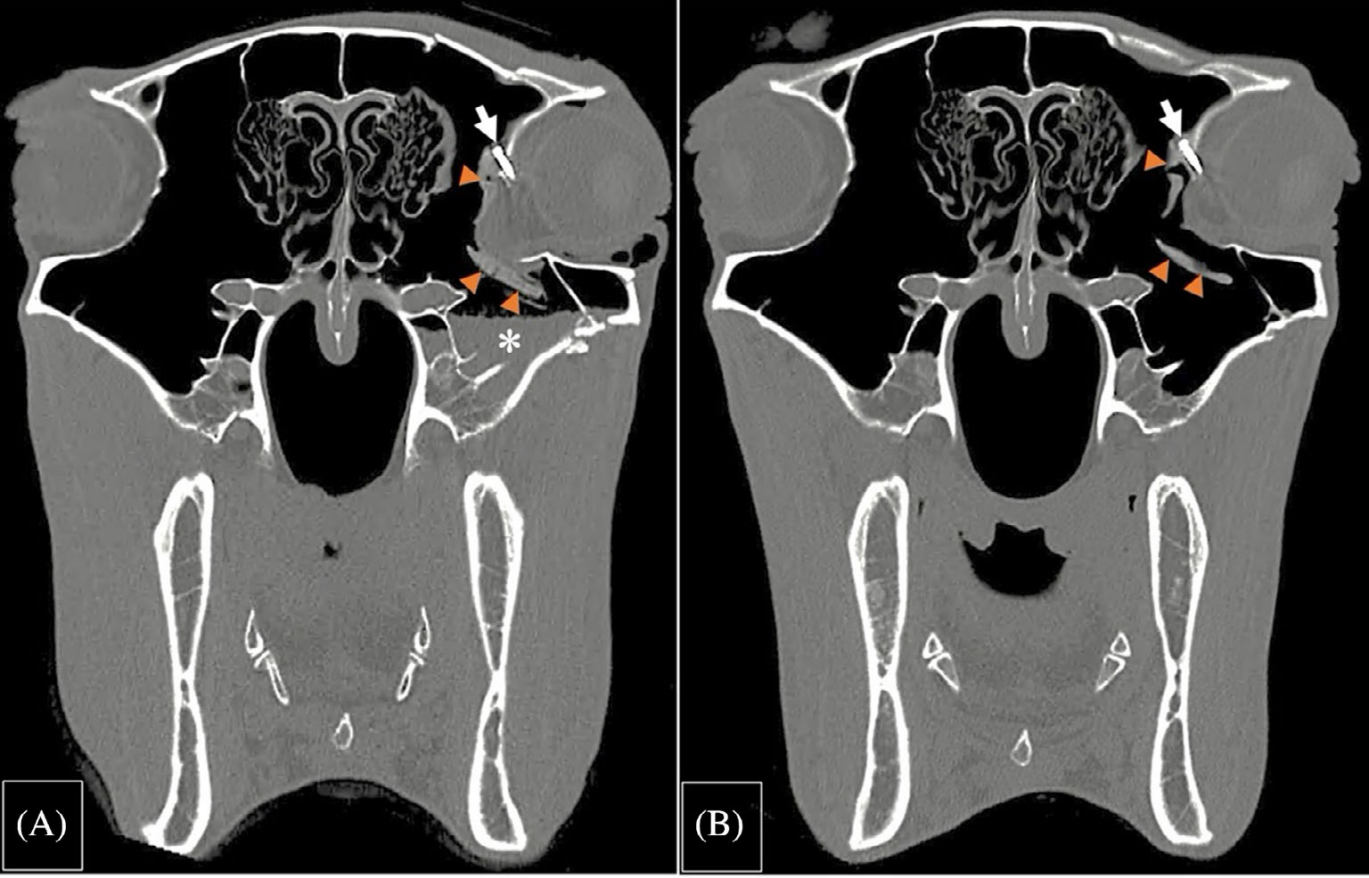
(A) First postoperative computed tomography (CT) scan, 6 days after surgery. The patient‐specific implant (PSI) is marked with arrowheads, and the 3.0 mm locking screw with a white arrow. A fluid line is visible in the left caudal maxillary and sphenopalatine sinuses (*). (B) Follow‐up CT scan, 11 weeks after surgery. The bone flap and fracture lines around the facial crest show progressive healing. Emphysema of the left eyelids has resolved completely. The PSI is marked with arrowheads, and the 3.0 mm locking screw with a white arrow.

**FIGURE 7:**
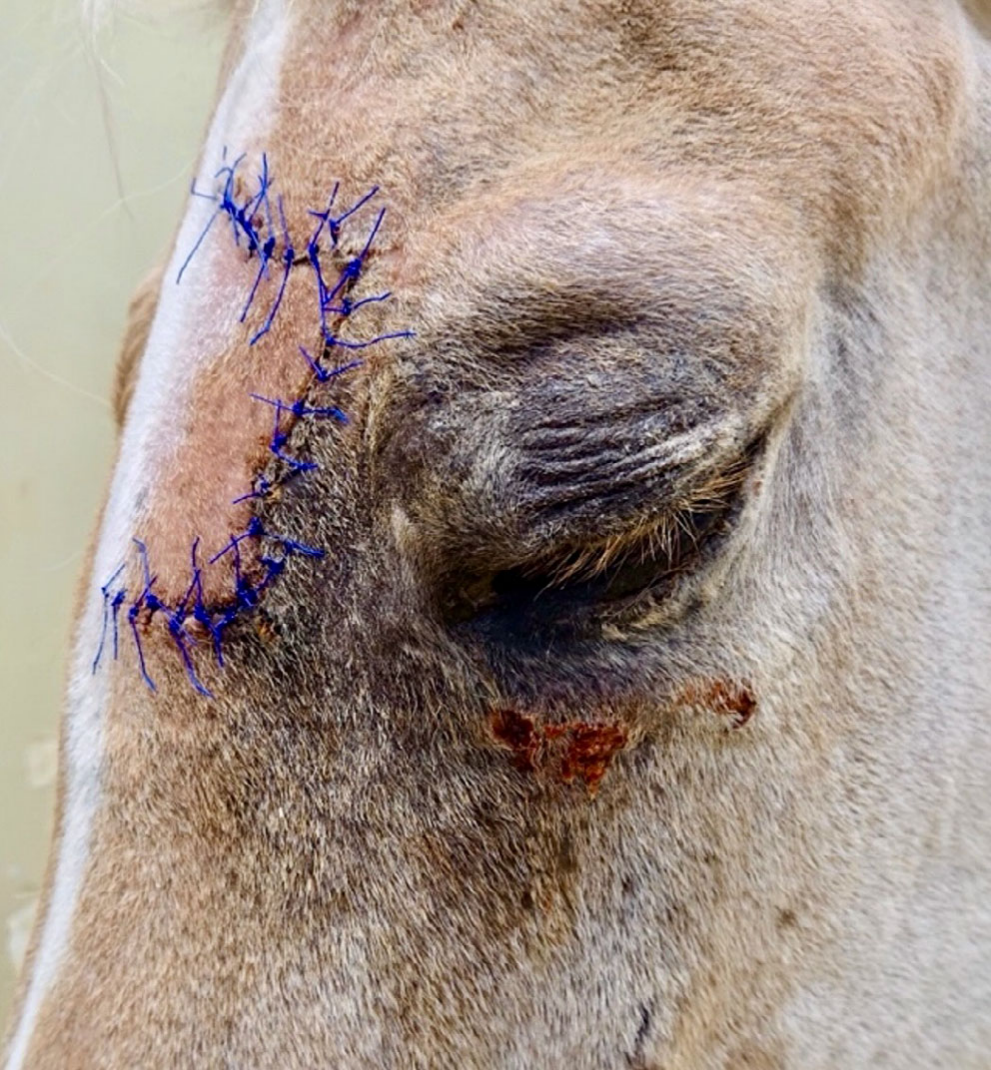
Photograph taken 9 days after surgery. The skin incision of the bone flap healed without complication. The skin over the toggle suture sites shows mild infection, with localized granulation tissue formation. Emphysema resolved, but ptosis of the left eye is visible. The client has given their consent for the photographs to be published.

Postoperatively, no emphysema was detected in the left upper or lower eyelid. Ptosis of the left eye was noted postoperatively (Figure 6). The mare was bright, alert, and responsive, with all vital parameters within normal limits. On the second postoperative day, mild serous epiphora was observed in the left eye. Fluorescein staining (Ophtorescein 5 mg/mL, CP‐Pharma) revealed two small, superficial corneal defects, each approximately pinhead‐sized, located near the medial canthus. The cornea was treated topically four times daily with an antibiotic ophthalmic ointment containing chlortetracycline hydrochloride, alternated with a vitamin A and dexpanthenol ophthalmic ointment administered at different time points during the day (Cepemycin, CP‐Pharma and Vitamycin, CP‐Pharma). Five days after surgery, fluorescein staining of the left cornea was negative. At the same time point, mild serous exudation was observed at the skin sutures overlying the buttons. These areas were cleaned twice daily. A CT scan of the head on postoperative day 6 showed that the implant was in situ but not fully in contact with the orbital wall, with slight retrobulbar soft tissue protrusion toward the paranasal sinus. Moderately attenuating material, with the appearance of a fluid line was visible in the left caudal maxillary sinus. The skin sutures above the buttons were removed on postoperative day seven to facilitate healing by secondary intention (Figure 7). Antimicrobial and anti‐inflammatory therapy was continued for 9 days (25 mg/kg BW sulfadiazine, 5 mg/kg BW trimethoprim orally, Sulfadimethoxin + Trimethoprim 50%, Serumwerk Bernburg AG, Bernburg, Germany every 12 h; 1.1 mg/kg BW flunixin‐meglumine orally, Flunidol, CP‐Pharma every 12 h). The skin sutures of the bone flap were removed 14 days postoperatively. The mare was discharged 19 days after surgery with normal vital parameters, unimpaired vision, and no signs of recurrent upper and lower eyelid emphysema or chemosis.

The horse was presented for a follow‐up CT scan 11 weeks after surgery. At that time, minimal ptosis of the left eye was still present. Vision was normal and there was no ocular or nasal discharge. The skin wounds at the button sites showed progressive healing, although a thin crust of wound exudate and granulation tissue remained. Follow‐up CT images confirmed that the shell implant remained in place. Compared to the immediate postoperative CT, less retrobulbar soft tissue protruded into the left caudal maxillary sinus and no evidence of upper and lower eyelid emphysema or sinusitis was apparent (Figure 6). Very mild mucosal swelling was observed medial to the buttons. The caudal button had migrated through the bone into the maxillary sinus and was embedded in the mucosa. All fracture lines in the region of the left facial crest and bone flap showed rounding of bone edges and blunting of previously sharp fracture margins. At 18 months postoperatively, the owner reported being highly satisfied with both the cosmetic outcome and the horse's general condition, and no further complications were observed.

## 4 DISCUSSION

This case report presents the successful reconstruction of a complex left orbital fracture in a horse using a 3D‐printed PSI. The use of 3D‐printed implants has previously been reported in small animals. For example, in a canine case report, a one‐stage craniectomy and cranioplasty were performed using 3D‐printed craniotomy cutting guides, followed by reconstruction with a patient‐specific 3D‐printed polyetheretherketone cranial implant.^17^ This report highlights one of the main advantages of PSIs, namely the improved accuracy and precise anatomical fit that facilitates reconstruction. To the authors' knowledge, the described surgical technique has not been previously documented in equine surgery.

Assessment of the left globe was initially difficult due to the degree of soft tissue swelling surrounding the eye, as seen in previously reported cases.^6^, ^9^ The authors hypothesize that the upper and lower eyelid emphysema developed due to communication with the caudal maxillary sinus. Air likely became trapped within the eyelids as retrobulbar soft tissue herniated through the fractured inner and ventral orbital walls, acting as a one‐way valve.

Ultrasonographic and radiographic examinations were limited in their ability to diagnose the cause of the eyelid emphysema. Radiographs have been reported to provide limited information about internal structures such as the eyes, and incomplete visualization of areas such as the sinuses and nasal passages.^18^ CT examination was essential to show the complexity of multiple fracture lines and the communication with the caudal maxillary sinus.

In this case, the printed implant was superimposed on the fractured orbit. During surgery, the implant was placed axially to the orbit through the concho‐frontal sinus. In the literature, different approaches to repair orbital fractures are described. In the case report of McMaster et al., a resorbable cuttable fixation plate was placed to support the orbital floor.^9^ In addition, a breast implant sizer and tissue expander were inserted into the concho‐frontal sinus to maintain the inner wall and ventral floor.^9^ Another method is intrasinusidal bolstering using a Foley balloon catheter, as described by Gardner et al.^7^ Patient‐specific implants are already in use for orbital fractures in human medicine. In contrast to equine surgery, where orbital wall reconstruction with a PSI required an intrasinusal approach, implantation in human medicine is most commonly performed via a transconjunctival route.^10^, ^19^, ^20^ Habib et al. mentioned the term “lock‐and‐key” type fit. This means that the implants can be molded to match the unique shape of the bone and can even restore missing orbital volume.^10^, ^19^, ^21^

In this report, PLA was used to print the implant. The polymer is composed of lactic acid monomers, which gradually degrade in situ. A major advantage of using a PSI, as opposed to a tissue expander, is that it does not require surgical removal, unless complications such as implant‐related infection arise. This eliminates the need for a planned second surgery and reduces overall patient burden. The limited stability achieved with PLA implants compared to high‐performance polymers or metal is not a limitation in this specific application, as the PSI acted more as a cover than an implant bridging a fracture under load. However, PLA was selected due to its ease of use and the availability of low‐cost additive manufacturing, particularly when compared to metal or high‐performance polymers.

One of the primary concerns was implant placement into a non‐sterile anatomical site, increasing the risk of infection. However, given the functional integrity of the eye and the anatomical extent of the defect, enucleation was not considered a viable alternative. Moreover, it remains unclear whether enucleation alone would have adequately closed the orbital wall defect or whether an implant would still have been necessary. Owners were informed that in the event of implant‐related complications, surgical access would be required to remove the implant from the sinus once the fracture had consolidated and an air‐tight seal had been re‐established.

Porcine small intestinal submucosa is a watertight and airtight membrane composed of bioactive, naturally occurring extracellular matrix, which induces angiogenesis and incorporates well into tissue.^22^ The material was shown to be safe and effective for reconstruction of orbital wall defects in humans.^23^ It was also reported for horses for treatment of corneal defects and limb wounds.^24^, ^25^ Although the PSI can be precisely shaped, the complexity of the internal orbital anatomy, limited surgical access, and the need for an airtight seal meant that implant‐to‐bone contact alone was insufficient to resolve the emphysema. Therefore, SIS was chosen as an intermediate layer to compensate for some of the expected topographical inaccuracies between the PSI and the reconstructed orbital wall. Moreover, it served to demonstrate that airtight coverage of the defect with a biologically compatible membrane can effectively eliminate orbital emphysema.

A limitation of this case report is that the authors can only present a single case using this technique, which limits the conclusions that can be drawn. The ventral attachment of the implant using knots over a metallic button resulted in mild infection at the suture sites, which were allowed to granulate by second intention (Figure 7). The caudal button even passed through the bone into the caudal maxillary sinus, but became trapped in the mucosa, which may have prevented the implant from moving. A fixation using the knotless TightRope fixation system (Arthrex GMBH) could have reduced the risk of skin dehiscence by eliminating the bulky knot of synthetic fiber under the skin. We would have preferred ventral screw fixation to eliminate the synthetic sutures as an additional source of complication, but screw placement was not considered possible due to the fracture lines around the facial crest and inadequate access ventral to the PSI.

**FIGURE 8:**
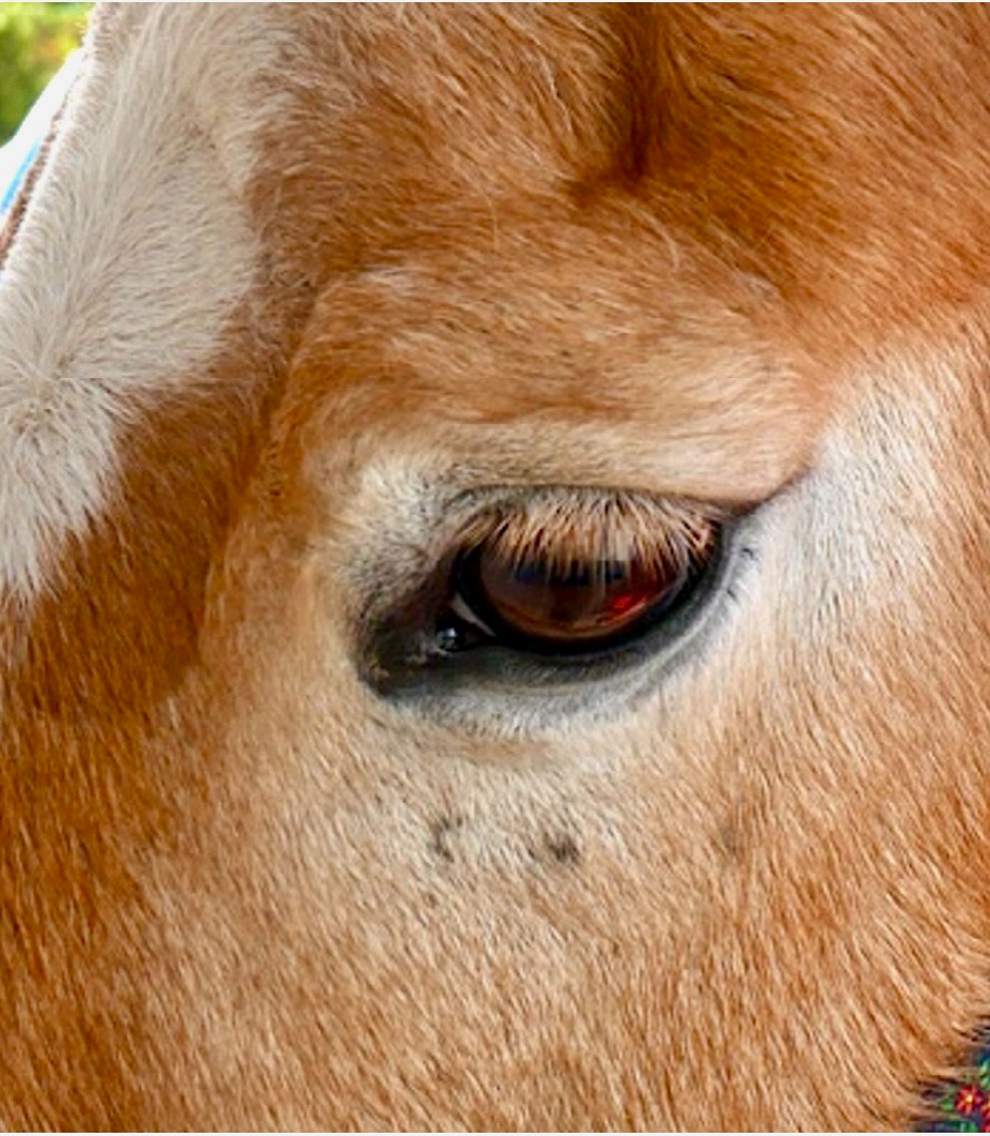
Postoperative photographs taken 18 months after surgery showed a favorable cosmetic outcome. Although minimal ptosis of the left upper eyelid is still noticeable, the overall appearance is positive. The client has given their consent for the photographs to be published.

A follow‐up CT scan at 11 weeks postoperatively, along with owner‐reported observations up to 18 months, revealed no evidence of infection or implant‐related complications. Only minor cosmetic asymmetry persisted, with a slightly narrower palpebral fissure on the affected side. Mild skin thickening over the subcutaneous suture buttons was observed but did not lead to any clinical concern (Figure 8).

Despite the limitations inherent in a single‐case report and the absence of a comparable conservatively treated case, this outcome supports the hypothesis that PSIs, shaped to reflect patient specific anatomy, hold substantial promise for treating complex orbital fractures in horses. Future clinical reports and comparative studies are needed to further evaluate the potential and limitations of this approach.

## AUTHOR CONTRIBUTIONS

Gernhardt J, DVM: Contributed to the design of the report, assisted surgical management, compiled all data, drafted, and revised manuscript. Böttcher P, DVM, DECVS: Contributed to the design of the report, design and development of the patient specific implant, scientific support in surgical management and provided scientific, in‐line editing of the manuscript. Eule JC, DVM, DECVO: Ophthalmological management of the case, scientific support in surgical management, provided intraoperative photographs, and provided scientific, in‐line editing of the manuscript. Mählmann K, DVM, DECVS: Oversaw data collection, provided scientific, in‐line editing of the manuscript. Müller E, DVM: Pre‐ and postoperative management of the case. Responsible for follow‐up examination. In‐line editing of the manuscript. Lischer CJ, DVM, DECVS: Contributed to the design of the report, was responsible for the surgical management, oversaw data collection, provided intraoperative photographs, interpreted data, and provided scientific, in‐line editing of the manuscript. All authors provided a critical review of the manuscript and endorse the final version. All authors are aware of their respective contributions and have confidence in the integrity of all contributions.

## CONFLICT OF INTEREST STATEMENT

The authors declare no conflicts of interest related to this report.
